# Successful Left Trisectionectomy for Intrahepatic Cholangiocarcinoma in a Patient with a Right-Sided Round Ligament: A Case Report

**DOI:** 10.70352/scrj.cr.24-0054

**Published:** 2025-02-22

**Authors:** Masashi Utsumi, Masaru Inagaki, Koji Kitada, Naoyuki Tokunaga, Koki Omoto, Naoki Onoda, Kosuke Yunoki, Hiroki Okabayashi, Ryosuke Hamano, Hideaki Miyaso, Yosuke Tsunemitsu, Shinya Otsuka, Rika Omote

**Affiliations:** 1 Department of Surgery, NHO Fukuyama Medical Center, Fukuyama, Hiroshima, Japan; 2 Department of Pathology, NHO Fukuyama Medical Center, Fukuyama, Hiroshima, Japan

**Keywords:** right-sided round ligament, intrahepatic cholangiocarcinoma, hepatic resection

## Abstract

**INTRODUCTION:**

A right-sided round ligament (RSRL) is a rare congenital anomaly characterized by the umbilical vein being connected to the right paramedian trunk. As it is associated with intrahepatic vascular anomalies, it poses special difficulties in hepatic resection, and an accurate understanding of those anomalies is indispensable.

**CASE PRESENTATION:**

An 80-year-old man visited a health clinic with the chief complaint of jaundice. Hyperbilirubinemia and impaired liver function were detected upon laboratory examination. Therefore, the patient was referred to our hospital for further examination and treatment. Contrast-enhanced computed tomography (CT) demonstrated dilatation of the intrahepatic bile ducts and the presence of a hypovascular tumor of 30 mm in size in the left lateral segment of the liver. The anterior branch of the portal vein (PV) formed a right-sided umbilical portion of the PV and was connected to the round ligament. This anomaly is known as an RSRL. The round ligament was located to the right of the gallbladder. Three-dimensional (3-D) CT of the PV clearly illustrated the independent ramification of the posterior branch and the subsequent bifurcation of the anterior branch and the left PV. Endoscopic, nasogastric biliary drainage was performed to treat the patient for obstructive jaundice, and endoscopic retrograde cholangiopancreatography demonstrated severe stenosis of the hilar bile duct. Biopsies of the stenotic bile ducts were suggestive of adenocarcinoma. The root of the posterior branch of the bile duct was intact from the cancer. The preoperative diagnosis was intrahepatic cholangiocarcinoma (T4N0M0, stage III B), according to the *American Joint Committee on Cancer Staging System*, 8th edition. Left trisectionectomy with extrahepatic bile-duct resection and hepaticojejunostomy was performed. The histological diagnosis of the tumor was intrahepatic cholangiocarcinoma (large duct type, 5.5 × 4.5 cm). The final pathological stage was T4N1M0, stage 3B. Three months after surgery, the patient was doing well without recurrence.

**CONCLUSIONS:**

The anatomy of patients with an RSRL should be evaluated in detail before surgery, especially when curative hepatic resection is performed for intrahepatic or perihilar cholangiocarcinoma.

## Abbreviations

ant-PV
: anterior branch of the portal vein
CT
: computed tomography
PV
: portal vein
post-PV
: posterior branch of the PV
RSRL
: right-sided round ligament

## INTRODUCTION

A right-sided round ligament (RSRL) is a rare congenital anomaly characterized by the connection of the umbilical vein to the right paramedian trunk, with a reported frequency of 0.1%–1.2%.^1,2)^ As it is associated with intrahepatic vascular anomalies, it causes special difficulties in hepatic resection, and an accurate understanding of those anomalies is indispensable. Here, we report a case of intrahepatic cholangiocarcinoma with an RSRL in which we performed left trisectionectomy.

## CASE PRESENTATION

An 80-year-old man visited a health clinic with the chief complaint of jaundice. Hyperbilirubinemia and impaired liver function were detected upon laboratory examination. Therefore, the patient was referred to our hospital for further examination and treatment.

Laboratory examination revealed that the leukocyte and C-reactive protein levels were within the normal range but that serum levels of total bilirubin (9.3 mg/dL; normal range, 0.2–1.2 mg/dL), aspartate aminotransferase (111 U/L; normal range, 13–33 U/L), alanine aminotransferase (301 U/L; normal range, 8–42 U/L), alkaline phosphatase (232 U/L; normal range, 115–359 U/L), gamma-guanosine triphosphate (617 U/L; normal range, 11–58 U/L), carcinoembryonic antigen (17.8 ng/mL; normal range, 0–5 ng/mL), and carbohydrate antigen 19-9 (145.9 U/mL; normal range, 0–37 U/mL) were high. The indocyanine-green retention rate 15 min after injection was 7.3%. The patient’s Child–Pugh classification was class A. Test results for hepatitis B surface antigen and antibodies against hepatitis C virus were negative.

**Fig. 1:**
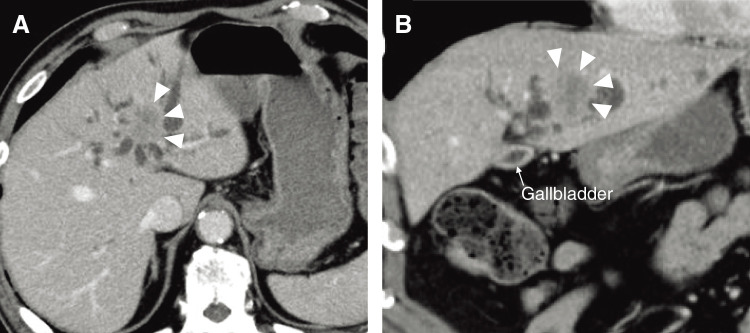
Contrast-enhanced computed tomographic images of the patient. (**A**) Axial section image. (**B**) Coronal section image. Dilatation of the intrahepatic bile ducts, a tumorous lesion, and a hypovascular tumor of 30 mm in size in the left lateral segment of the liver (arrowheads).

**Fig. 2:**
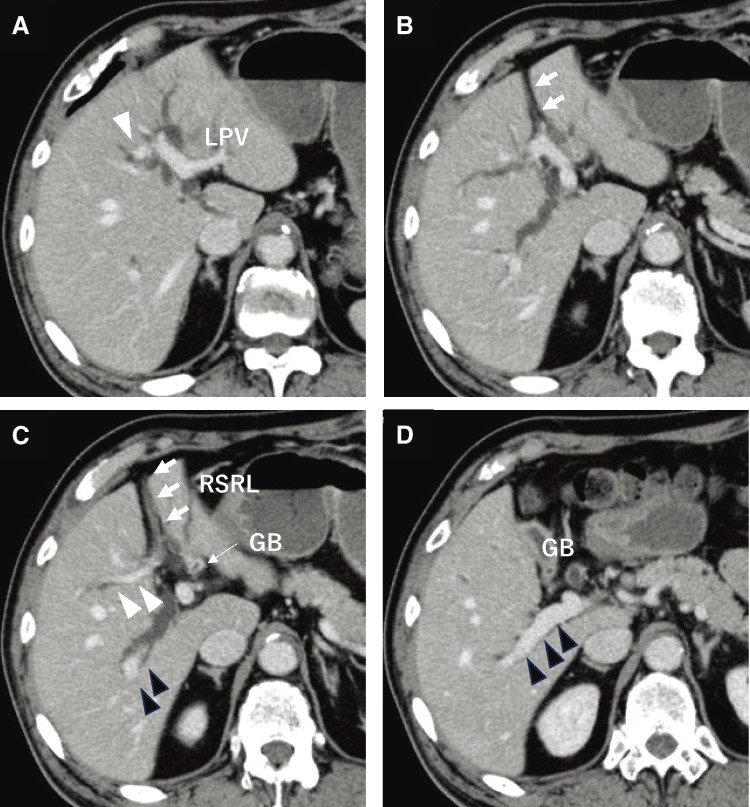
Contrast-enhanced computed tomographic images of the patient. The posterior branch of the PV (black arrowheads) is ramified independently (Ppost-i type, D), followed by the ramification of the anterior branch of the PV (white arrowheads) and the LPV (**A–C**). The ant-PV formed a right-sided umbilical portion of the PV and was connected to the round ligament (white arrows) (**B**, **C**). The RSRL (white arrows) was located to the right of the gallbladder (**C**, **D**). ant-PV, anterior branch of the portal vein; GB, gallbladder; LPV, left portal vein; PV, portal vein; RSRL, right-sided round ligament

**Fig. 3:**
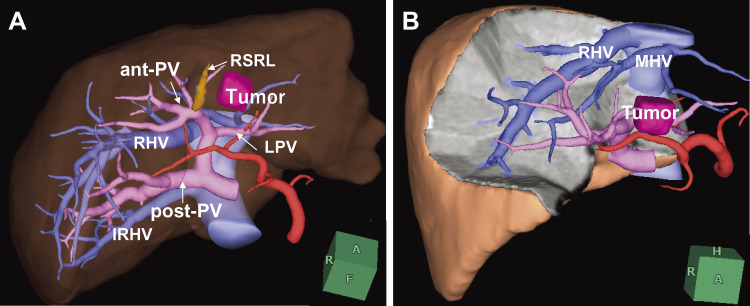
3-D CT images of the patient. (**A**) The image clearly illustrates the independent ramification of the post-PV and the subsequent bifurcation of the ant-PV and LPV. (**B**) 3-D CT images were captured using a Synapse Vincent 3-D volume analyzer (Fujifilm Holdings Corporation, Tokyo, Japan). ant-PV, anterior branch of the portal vein; IRHV, inferior right hepatic vein; LPV, left portal vein; MHV, middle hepatic vein; post-PV, posterior branch of the portal vein; RSRL, right-sided round ligament; 3-D CT, 3-dimensional computed tomography

Contrast-enhanced computed tomography (CT) demonstrated dilatation of the intrahepatic bile ducts and the presence of a hypovascular tumor of 30 mm in size in the left lateral segment of the liver (**Fig. 1**). The posterior branch of the portal vein (post-PV) ramified independently from the main portal trunk (Ppost-i type), followed by ramification of the anterior branch of the PV (ant-PV) and the left PV (LPV) (**Fig. 2**). The ant-PV formed a right-sided umbilical portion of the PV and was connected to the round ligament (**Fig. 2**). The round ligament was located to the right of the gallbladder (**Fig. 2**). 3-D CT of the PV clearly illustrated the independent ramification of the posterior branch and the subsequent bifurcation of the ant-PV and LPV (**Fig. 3**). 3-D CT images were captured using a Synapse Vincent 3-dimensional volume analyzer (Fujifilm Holdings Corporation, Tokyo, Japan).

**Fig. 4:**
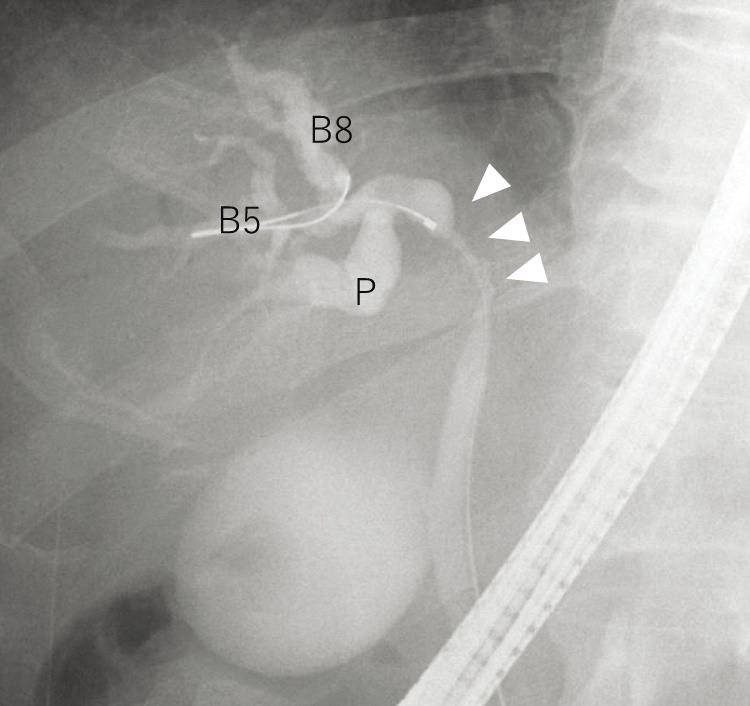
Endoscopic cholangiopancreatography of the patient. Severe stenosis of hilar bile duct (arrowheads). The stenotic portion extended from the hepatic hilum to the right (the B2 and B3 bile ducts). P, posterior branch of hepatic bile duct

**Fig. 5:**
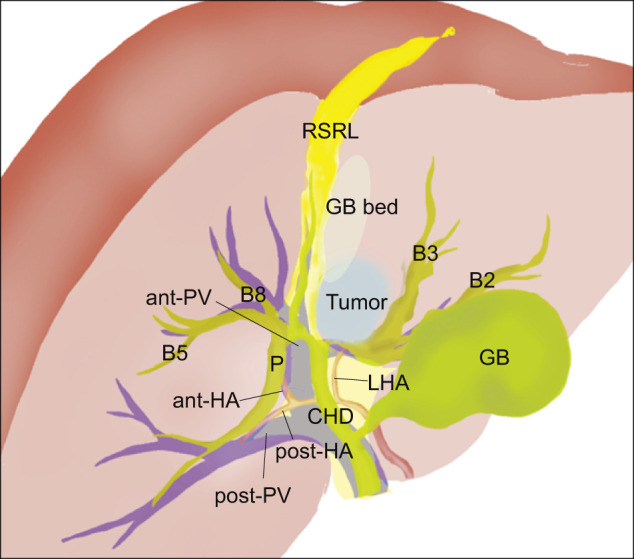
Preoperative schema of perihilar anatomy and cancer position. ant-HA, anterior branch of the hepatic artery; ant-PV, anterior branch of the portal vein; CHD, common hepatic duct; GB, gallbladder; LHA, left hepatic artery; P, posterior branch of the hepatic bile duct; post-HA, posterior branch of the hepatic artery; RSRL, right-sided round ligament

Endoscopic, nasogastric biliary drainage was performed to treat the patient for obstructive jaundice, and endoscopic retrograde cholangiopancreatography demonstrated severe stenosis of the hilar bile duct (**Fig. 4**). The stenotic portion extended from the hepatic hilum to the right, B2, and B3 bile ducts (**Fig. 4**). The preoperative schema of the perihilar anatomy and cancer progression is shown in **Fig. 5**. Biopsies of the stenotic bile ducts were suggestive of adenocarcinoma. The root of the posterior branch of the bile duct was not infiltrated by the cancer. According to CT volumetry, the volume of the posterior section of the liver was 610 mL (50.7% of the total liver volume). Positron emission tomography revealed focally increased glucose uptake in the tumor, suggesting malignancy. The preoperative diagnosis was intrahepatic cholangiocarcinoma (T4N0M0, stage III B).^3)^

### Operation

**Fig. 6:**
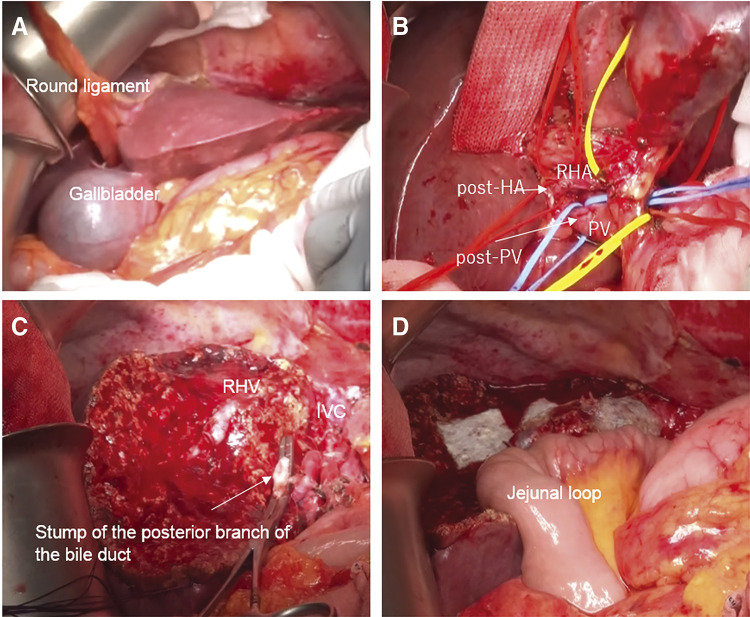
Operative observations. (**A**) The gallbladder is located to the left of the round ligament. (**B**) The hepatic hilum was dissected, and the vessels were wrapped with tape. (**C**) The dissected surface after left trisectionectomy. Liver resection was performed along the RHV. (**D**) Biliary reconstruction was performed using the Roux-en-Y method. IVC, inferior vena cava; post-HA, posterior branch of hepatic artery; post-PV, posterior branch of the portal vein; PV, portal vein; RHA, right hepatic artery; RHV, right hepatic vein

A left trisectionectomy with extrahepatic bile-duct resection and hepaticojejunostomy was performed (**Fig. 6**). The left, right anterior, and posterior arteries, the common hepatic duct, and the root of the LPV, ant-PV, post-PV were sequentially dissected and taped. After clamp testing to confirm inflow into the posterior lesion, the left and right anterior arteries and the root of the LPV and ant-PV were ligated and divided. Thereafter, the posterior branch of the bile duct was divided and taped. Hepatic parenchymal resection, guided by a demarcation line, was conducted along the right hepatic vein, and the common trunk of the middle and left hepatic vein was divided. Finally, the posterior branch of the bile duct was separated, and the specimen was removed. The result of the frozen section analysis of the bile-duct stump was negative for carcinoma (on both the duodenal and hepatic sides). Biliary reconstruction was performed using the Roux-en-Y method. The operation time was 7 h 30 min, and the intraoperative blood loss was 750 mL.

**Fig. 7:**
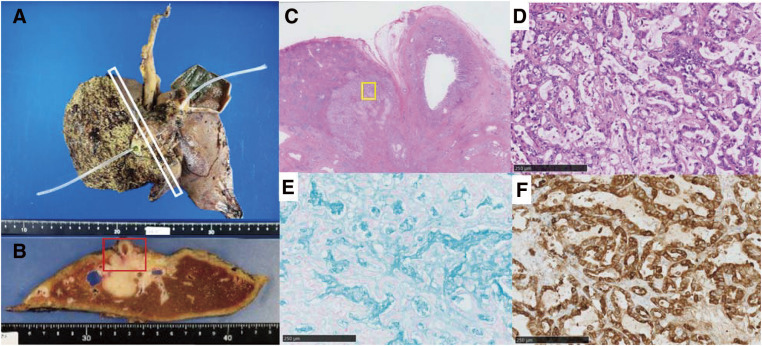
Histopathological examination. (**A**) Gross photograph of the resected specimen. (**B**) Cut surface in the white square of panel (**A**). It contained a solid whitish mass measuring 5.5 × 4.5 cm with irregular margins. (**C**) Hematoxylin and eosin staining of the area in the red square in panel (**B**). (**D–F**) The area in the yellow square in panel (**C**). (**D**) Hematoxylin and eosin staining. Columnar tumor cells, poorly differentiated and arranged in irregular glandular structures. (**E**) Alcian blue staining. The tissue stained positively, representing mucus-secreting cells or intraluminal mucus. (**F**) Epithelial membranous antigen staining. The tissue stained diffusely positive.

The histological diagnosis of the tumor was intrahepatic cholangiocarcinoma (large duct type, 5.5 × 4.5 cm) (**Fig. 7**). Metastasis was detected in 5/11 regional lymph nodes. The final pathological stage was T4N1M0, stage 3B, according to the 8th edition of the *American Joint Committee on Cancer Staging System*.^3)^ An intra-abdominal abscess developed after the operation. We treated the patient with drainage of the abscess cavity and with antibiotics, and he was discharged 25 days after surgery. He declined adjuvant chemotherapy. Three months after surgery, the patient was doing well without recurrence.

## DISCUSSION

This is the first report to describe a major hepatectomy for curative resection of intrahepatic cholangiocarcinoma in a patient with an RSRL. An RSRL arises as a result of the diminishing of the left umbilical vein during the prenatal period. Consequently, the right umbilical vein forms the umbilical portion of the PV.^4)^ Anomalies associated with an RSRL may lead to several surgical problems during hepatectomy. Therefore, the type of anomaly should be determined during hepatectomy planning for patients with an RSRL.^4–6)^ Shindoh et al.^5)^ and Nishitai et al.^6)^ classified portal, arterial, and biliary ramifications, as discussed below.

PV ramifications of an RSRL can be classified into 3 types: the Ppost-i, P-bifurcation, and P-trifurcation types.^5)^ In the Ppost-i type, the posterior branch of the PV ramifies independently of the main portal trunk. In the P-bifurcation type, the main PV ramifies into the right PV and the LPV, similar to the normal anatomy, whereas, in the P-trifurcation type, the main PV ramifies concurrently into the left, anterior, and posterior branches. In the present case, the ramification pattern of the main portal branch was of the Ppost-i type.

Hepatic arterial ramifications of the RSRL are classified into 3 types: independent ramification of the left hepatic artery, common trunk formation between the left hepatic artery and the ventral branch of the right paramedian arteries, and replacement of the left hepatic artery by the left gastric artery.^6)^ The ramification in our case was classified as independent. Finally, intrahepatic bile-duct confluence patterns are classified into 4 types: symmetrical, independent right lateral, total left, and total right.^6)^ The pattern in our study corresponded to the total-left type. In this pattern, the entire right hemiliver is drained via a thick biliary branch that is connected to the left biliary branch and passes across the umbilical fissure towards the left and backward toward the right umbilical portions.^6)^

No specific relationship has been reported among the patterns of biliary, portal, and arterial ramifications in livers with an RSRL.^6)^ In patients with an RSRL, the proportions of the liver differ from those of a normal liver.^5,7)^ In livers with an RSRL, each side of the lateral sectors (the S2 and posterior sections) and the dorsal portion of the right paramedian sector are reportedly larger than those of typical livers, whereas the left paramedian sector (S3 + S4) and the ventral portion of the right paramedian sector are reportedly substantially smaller.^5)^ Ishida et al. proposed the following explanation for this observation: as the umbilical vein is connected to the right side of the liver, the right side of the liver may shift to the left and become enlarged, whereas the left side of the liver may shrink.^8)^

In one population of individuals with normal (left-sided) round ligaments, the posterior section of the Ppost-i type anatomy was significantly larger than that of patients with a P-bifurcation anatomy.^7)^ In the present case, the ramification pattern of the main portal branch was the Ppost-i type, and its posterior section was relatively large. Ishida et al. reported that left trisectionectomy has several surgical advantages, especially in patients with cholangiocarcinoma associated with a Ppost-i-type RSRL.^8)^ First, a large liver-remnant volume can be retained. Second, ligation and division of the ant-PV and LPV are easier in such cases. Third, bile-duct division and reconstruction can be performed in the usual manner because the biliary anatomy at the root of the posterior Glissonean pedicle is distant from the tumor, which is close to the complicated anatomy of the hepatic hilum associated with the RSRL. Although left trisectionectomy is a technically demanding operation with a relatively high mortality rate,^9,10)^ it has become one of the standard procedures for perihilar cholangiocarcinoma at high-volume centers.^11,12)^ Ishida et al. reported on a case of left trisectionectomy for perihilar cholangiocarcinoma with an RSRL and described that, in patients without invasion of the posterior section, left trisectionectomy may be favorable.^8)^ Therefore, left trisectionectomy may also be favorable for patients with intrahepatic cholangiocarcinoma close to the hilum with anomalous patterns of RSRL, as in our case, as long as the tumor does not extend into the posterior section.

Reports over the last decade on liver resection for malignant hepatobiliary tumors in patients with an RSRL are summarized in **Table 1**.^8,13–16)^ We summarized 12 cases, including our 3 cases (this case and 2 prior cases). In all cases, an RSRL and an anomaly of vascular ramification were observed upon preoperative CT. Modified hepatectomy was performed in all of those previous studies, according to the portal, arterial, and biliary ramifications, without major complications.

**Table 1:** Clinicopathological characteristics and surgical procedures of patients with a right-sided round ligament

Author	Year	Sex	Disease	Operation	Portal anatomy	Biliary anatomy	Postoperative complication
Almodhaiberi et al.^13)^	2015	Male	CCC	Left hemihepatectomy	Ppost-i	Symmetrical type	None
Hai et al.^14)^	2017	Male	CCC	Extended left hemihepatectomy	Ppost-i	Total-left type	Bile leakage
Goto et al.^15)^	2018	Male	GBC	Right hemihepatectomy	Trifurcation	Independent right-lateral type	None
Terasaki et al.^16)^	2019	Male	CRLM	Lap-left lateral sectionectomy	Bifurcation	Symmetrical type	None
Ishida et al.^8)^	2020	Male	HCC	Partial hepatectomy	Ppost-i	NA	None
Ishida et al.^8)^	2020	Male	HCC	Partial hepatectomy	Ppost-i	NA	None
Ishida et al.^8)^	2020	Female	CRLM	Right hemihepatectomy	Bifurcation	NA	None
Ishida et al.^8)^	2020	Male	CCC	Left trisectionectomy	Ppost-i	Total-left type	None
Ishida et al.^8)^	2020	Male	HCC and CCC	Extended posterior sectionectomy	Bifurcation	Symmetrical type	None
Our previous case	2022	Female	GBC	Partial hepatectomy	Ppost-i	Symmetrical type	None
Our previous case	2022	Male	CRLM	Segmentectomy	Bifurcation	Total-left type	None
Our current case	2024	Male	ICC	Left trisectionectomy	Ppost-i	Total-left type	Abdominal abscess

CCC: perihilar cholangiocarcinoma; CRLM: colorectal liver metastasis; GBC: gallbladder carcinoma; HCC: hepatocellular carcinoma; ICC: intrahepatic cholangiocarcinoma: Lap: laparoscopy: NA: not available

Preoperative liver simulations have demonstrated the characteristics of ideal simulation and navigation tools.^17)^ Simulations can accurately predict the liver volume to be resected and permit stereotactic measurement of the length of the surgical margin. In the present case, the most useful contribution of liver simulation was the visualization of the volume and 3-D structure of the intrahepatic vasculature. 3-D simulations based on precise intrahepatic vascular and biliary analyses enable accurate and oncologically curative hepatic resection, even in patients with rare anatomical anomalies.

## CONCLUSIONS

The vascular and biliary anatomy of patients with an RSRL is complicated and should be evaluated in detail before surgery by using a hepatectomy simulation system, especially when curative hepatic resection is performed for intrahepatic or perihilar cholangiocarcinoma.

## DECLARATIONS

### Funding

The authors declare that they received no funding for this study.

### Authors’ contributions

MU drafted the manuscript and provided the original figures.

MI critically reviewed and revised the manuscript.

KK and NT collected the clinical and radiological data.

KO, NO, KY, HO, RH, HM, YT, and SO reviewed the manuscript.

RO provided pathological data.

All authors read and approved the final manuscript.

### Availability of data and materials

The data generated and/or analyzed in this study will be made available upon reasonable request.

### Ethics approval and consent to participate

Not applicable.

### Consent for publication

Informed consent to publish the case details was obtained from the patient.

### Conflicts interest

None of the authors have conflicts of interest to report.
